# Chronic Conditions, Multimorbidity and Their Associations With Tooth Loss, Dental Caries and Periodontal Disease: An Australian Perspective

**DOI:** 10.1155/ijod/6649238

**Published:** 2026-09-09

**Authors:** Najith Amarasena, Manisha Tamrakar, Sergio Chrisopoulos, Gloria Mejia, Liana Luzzi

**Affiliations:** ^1^ Australian Research Centre for Population Oral Health, School of Dentistry, College of Health, Adelaide University, Adelaide 5005, South Australia, Australia, adelaide.edu.au; ^2^ Department of Paediatric Dentistry and Dental Public Health, Adams School of Dentistry, University of North Carolina at Chapel Hill, Chapel Hill, North Carolina, USA, unc.edu

**Keywords:** chronic conditions, dental caries, dentistry, oral health, periodontal disease, tooth loss

## Abstract

**Introduction:**

There is a paucity of nationwide population‐based studies exploring the associations between chronic conditions and oral health. This study was conducted to describe the sociodemographic characteristics of a sample of Australian dentate adults with chronic conditions and to ascertain the associations of chronic conditions with tooth loss, dental caries and periodontal disease.

**Methods:**

Data were drawn from the National Study of Adult Oral Health (NSAOH) 2017–18, which included a random sample of Australians aged 15+ years. NSAOH used online/telephone interviews to elicit self‐reported information, including sociodemographic characteristics, and oral examinations to collect clinical data. Information on chronic conditions was collected in the pre‐examination questionnaire. Clinical information was ascertained in 5022 dentate adults. A 95% confidence interval (95% CI) was considered for proportions and means calculations.

**Results:**

While age was strongly associated with chronic conditions, their distribution showed a social gradient linked to education and income. Individuals with chronic obstructive pulmonary disease (COPD) (mean number of missing teeth = 11.5 [95% CI = 8.3, 14.7]) and kidney disease (mean number of missing teeth = 11.3 [95% CI = 8.5, 14.1]) had higher tooth loss whereas dental caries experience was higher in people with heart disease (mean number of decayed tooth surfaces [DS] = 2.5 [95% CI = 1.2, 3.7]) and depression (mean number of DS = 2.3 [95% CI = 1.4, 3.3]). Gingivitis was more prevalent in individuals with diabetes (40.1% [95% CI = 31.5, 49.4]) and high blood pressure (33.9% [95% CI = 29.3, 38.9]) while adults with heart disease (59.1% [95% CI = 39.3, 76.3]) and cancer (56.7% [95% CI = 43.2, 69.3]) had higher levels of periodontitis. Individuals with multimorbidity (presence of two or more concurrent chronic conditions) experienced greater tooth loss, dental caries and periodontitis.

**Conclusions:**

Australian dentate adults—particularly those with multimorbidity—exhibit significantly worse oral health statuses than healthy counterparts. These findings highlight the critical need to integrate oral care into general health services and implement targeted dental programmes for populations with chronic illnesses.

## 1. Background

Despite being preventable and controllable, oral diseases including untreated dental decay, severe periodontitis and edentulism are the most prevalent chronic conditions affecting around 3.69 billion people [1]. The World Health Organization (WHO) has reported that untreated dental decay of permanent dentition is the most ubiquitous condition: severe periodontitis and edentulism rank next, afflicting 2 billion, 1 billion, and 350 million cases, respectively, whereas the estimated global prevalence of these conditions is 29%, 19% and 7%, respectively [2]. Among chronic conditions, oral diseases ranked first and third in the world, for prevalence and incidence, in 2019 [3], regardless of the World Bank country income profile, with an average global prevalence of 45% [2] and an age‐standardised incidence rate (ASIR) of 57,481 per 100,000 people [4]. According to WHO, the global burden of oral diseases exceeds the combined next five most prevalent non‐communicable diseases (mental disorders, cardiovascular disease, diabetes mellitus, chronic respiratory diseases and cancers) by nearly a billion cases [2]. In Australia, the proportion of dentate adults aged 15+ years with untreated coronal decay was 32%, while that of moderate to severe periodontitis and edentulism was 30% and 4%, respectively [5]. The contribution of oral disorders to the total (fatal + non‐fatal) burden of disease and non‐fatal burden of disease in Australia was 2.3% and 4.2%, respectively, in 2023 [6]. It was noteworthy that the total burden of oral diseases in Australia increased by 3.6% between 2003 and 2023, while that of all disease groups in combination decreased by 11.1% during the same period [6].

Oral health is considered fundamental to overall general health and well‐being, while oral diseases are a major public health problem [2]. Oral diseases share common causal pathways, social determinants and risk factors with chronic systemic conditions, which in turn may lead to co‐morbidity, the frequent co‐occurrence of both oral and chronic systemic conditions [2, 7, 8]. Chronic conditions/diseases, also known as long‐term health conditions/non‐communicable diseases, are ubiquitous both in Australia and worldwide and a significant concern at global, regional, national and individual levels [2, 8–11]. As per the latest WHO reports, the estimated number of cases globally for the most prevalent chronic conditions (not including oral diseases) is about 2.5 billion with mental disorders (967 million cases), cardiovascular diseases (522 million cases), diabetes mellitus (458 million cases), chronic respiratory diseases (453 million cases) and cancers (86 million cases) contributing to this estimate [2]. Chronic conditions account for nearly 74% of deaths globally, while over 80% of such premature deaths are attributed to four main groups of chronic conditions: cardiovascular diseases, cancer, chronic respiratory diseases and diabetes [9]. According to the Australian Bureau of Statistics (ABS), the most prevalent chronic conditions among Australians in 2022 (in descending order) are mental and behavioural conditions, back problems, arthritis, asthma, diabetes, cardiovascular diseases and stroke, osteoporosis, chronic obstructive pulmonary disease (COPD), cancer and kidney disease [10]. In 2022, the Australian Institute of Health and Welfare (AIHW) estimated that around 61% of Australians of all ages had at least one chronic condition, while coronary heart disease, dementia, back pain and problems, anxiety disorders and COPD were the five leading causes of disease burden in Australia [11]. It was also estimated that 38% of Australians had multimorbidity, the presence of two or more chronic conditions at the same time [11]. Moreover, 89%–92% of all deaths in Australia each year between 2002 and 2022 were attributed to chronic conditions [11].

Common chronic conditions that are regarded as linked with oral conditions are type 2 diabetes mellitus, cardiovascular diseases and cerebrovascular diseases [12]. Less frequent but also associated are COPD, cognitive impairment and dementia [12], rheumatoid arthritis, asthma, liver disease, ankylosing spondylitis [13, 14], chronic kidney disease and cancer (lung, head and neck and pancreas) [13]. Several researchers have suggested that chronic systemic conditions and oral diseases are correlated [12, 13]. Seitz et al. [12], in an umbrella review, found 107 correlations between oral diseases and systemic conditions. Another umbrella review in 2022 showed strong associations of oral diseases with 28 non‐communicable diseases including five types of cancers [13]. In both reviews, the most reported oral disease associated with chronic conditions was periodontitis [12–14], and other less frequently linked were tooth loss and dental caries [12, 14]. Periodontal disease alone has been hypothesised to be linked with 57 systemic conditions based on a systematic mapping of the WHO International Clinical Trials Registry Platform [14]. Further, two systematic reviews suggested that dental caries and tooth loss were higher among individuals with depressive disorder and anxiety [13, 15]. Dental caries and tooth loss have also been associated with pneumonia and certain cancers [2].

Australian adults with a chronic condition had reported more oral health impacts such as experiencing toothache and orofacial pain, feeling uncomfortable with their oral appearance and avoiding certain foods due to oral problems than their counterparts without a chronic condition [16]. Moreover, self‐reported oral health was worse and dental visiting patterns were less favourable among Australian adults with chronic conditions as opposed to individuals with no chronic conditions [17]. These findings were based on the National Dental Telephone Interview Survey of Australian residents in 2010. However, there has been a paucity of studies, particularly at the national level, exploring the associations between chronic conditions and oral health in Australia since then. Moreover, relatively few population‐based studies on chronic conditions‐oral health relationships are available in the literature, as reported by a recent umbrella review [13]. Whilst adding strength to the body of evidence on the associations between chronic conditions and oral health, findings from such studies are valuable for health care providers as well as policy makers to support oral health self‐care and integrate oral care into the overall health care of people with chronic conditions. This is particularly relevant given that a holistic approach to the simultaneous management of oral and general health among patients with chronic systemic conditions has often been neglected, with the emphasis being placed solely on managing their systemic conditions [8]. As such, the present study was carried out using the database of the National Study of Adult Oral Health (NSAOH 2017–18), which comprised the latest nationwide oral health information of Australian adults. The objectives of the study were to describe the sociodemographic characteristics of Australian dentate adults with chronic conditions and to ascertain the associations between chronic conditions, multimorbidity and oral diseases including tooth loss, dental caries and periodontal disease.

## 2. Methods

Previous reports have comprehensively described the study design and sample size calculation for NSAOH 2017–18 [5, 18, 19]. Briefly, parameter estimates, variances and design effects were generated using data from the previous National Survey of Adult Oral Health (NSAOH 2004–06). The minimum detectable thresholds for assessing changes in the prevalence and extent of oral diseases in the Australian adult population since the 2004–06 study were selected as a 10% change in estimates of mean decayed, missing or filled teeth (DMFT) and a 20% change in the prevalence of moderate or severe periodontitis. Type 1 and II errors were set at 0.05 and 0.20, respectively. It was assumed that 50% of the individuals would participate in the examination. Since edentulous participants would be interviewed but not examined, it was decided to increase the overall number of interviews for participants with and without teeth. Based on these calculations, a total sample size of 15,200 was targeted to achieve 7200 oral examinations [5]. Ethical approval for NSAOH 2017–18 was provided by the Human Research Ethics Committee (HREC) of the University of Adelaide.

A three‐stage stratified probability sampling design was employed to recruit a random sample of Australians aged 15 years and above from all Australian states and territories. After sampling postcodes within states/territories, persons aged 15 years and above were chosen within selected postcodes from the Medicare database by Services Australia, formerly known as the Australian Government Department of Human Services (DHS). Online or telephone (CATI [computer‐assisted telephone interview]) interviews were conducted by trained interviewers based on a questionnaire used in the past. Signed, informed consent was obtained from all examined participants aged 18+ years before conducting the examination, while parents/guardians provided signed, informed consent prior to the examination of participants aged 15–17 years. Information on sociodemographic characteristics and chronic conditions was obtained through interviews. Sociodemographic characteristics included in the current analysis were age (<45 years: ≥45 years), sex (male: female), indigenous identity (indigenous: non‐indigenous), residential location (major cities: remote/rural), the level of education (degree or higher: other/none) and income tertile (lowest [$40K]: middle [$40K–<$90K]: highest [$90K+]). Although NSAOH 2017–18 had obtained information on 16 chronic conditions, it was decided to include only 10 conditions (namely, heart diseases, diabetes, kidney disease, asthma, high blood pressure, stroke, COPD, arthritis/osteoporosis, cancer and depression) in the current analysis. The conditions excluded were haemophilia, individuals with pacemakers/defibrillators, artificial materials such as heart valves, hip joint replacement, organ transplantation and dental implants. Haemophilia was excluded due to the small number of cases (*n* = 8), while other conditions were considered too non‐specific to be classified as chronic conditions.

Oral examinations were carried out by 40 calibrated dental practitioners, adhering to a uniform procedure used by the public dental clinics of the pertinent state or territory dental health services. Participants had to consent to an oral examination. Replicate pairs of examinations with 101 study participants were conducted to assess inter‐examiner reliability relative to a gold standard examiner [5]. Kappa values for inter‐examiner reliability in assessing the prevalence of periodontal disease, dental caries and tooth loss were 0.64 and 0.96, 1.0, respectively, which indicated a substantial to almost perfect agreement [5]. The examiners conducted the oral examinations using regular dental chairs with an overhead light providing illumination. Moreover, an intra‐oral mirror with its own battery‐powered light was used to facilitate the detection of enamel caries during visual examination. The following guidelines and criteria were used in assessing tooth loss, dental caries and periodontal disease.

### 2.1. Tooth Loss

Complete tooth loss was assessed according to the answer to the interview question: ‘Do you have any natural teeth’? (response categories: ‘Yes’/’No’) [5, 19]. Existing crowns and caps were recorded as natural teeth, whereas dental implants were not. Missing teeth that had been removed due to dental caries or periodontitis and teeth missing due to any other reason for individuals aged less than 45 years were differentiated by the examiners. For participants aged 45+ years, the removed or absent tooth was recorded as missing. In the current analysis, tooth loss was indicated by the average number of missing teeth due to dental caries and periodontal disease per person and inadequate natural dentition (IND). The presence of fewer than 21 natural teeth in an individual was regarded as IND [5].

### 2.2. Dental Caries (Coronal Caries)

Dental caries was assessed on five tooth surfaces (mesial, buccal, distal, lingual and occlusal/incisal) by means of visual criteria without using an explorer. The mean number of decayed tooth surfaces (DS) per person and the mean number of decayed, missing or filled tooth surfaces (DMFS) per person were computed to indicate the severity of untreated dental caries (the burden of disease) and the severity of dental caries experience (lifetime experience of dental caries), respectively. DMFS implies lifetime experience of dental caries since structurally broken‐down enamel cannot be healed, and treating it by filling or pulling out leaves a permanent sign of disease [5, 18, 19].

### 2.3. Periodontal Disease

Periodontal disease was clinically assessed in participants with no medical contraindications for periodontal probing. The two types of periodontal disease, gingivitis and periodontitis, were ascertained based on the following criteria.

Gingivitis: inflammation of the marginal gingival tissues around six index teeth (if present: the most anterior molar tooth in each dental quadrant + right upper central incisor + left lower central incisor) was assessed using the gingival index of Loe and Silness [20].

Periodontitis: The US National Health and Nutrition Examination Survey (NHANES) methods were employed to record periodontal tissue destruction [21]. Three aspects (mesio‐buccal, mid‐buccal and disto‐buccal) of all teeth present, except third molars, were assessed using a periodontal probe (Hu‐Friedy PCP2, Chicago, Illinois). The prevalence of moderate and severe periodontitis was described according to the US Centers for Disease Control and Prevention (CDC) and the American Academy of Periodontology (AAP) case definition [22]. Accordingly, the following case definitions were adopted in the present study.

a. Moderate periodontitis: the presence of either at least two proximal sites, not on the same tooth, with attachment loss of 4 mm or more or at least two such sites that had pockets of 5 mm or more.
b. Severe periodontitis is the presence of at least two proximal sites, not on the same tooth, with attachment loss of 6 mm or more, plus at least one periodontal pocket with a depth of 5 mm or more.

### 2.4. Data Analysis

Representativeness of the target population was ensured by weighting all data to population benchmarks [18]. SAS software Version 9.4 (SAS 9.4; SAS Institute Inc., Cary, NC, USA) was employed to manage the data files and compute summary variables. A 95% confidence interval (95% CI) was considered for proportions and calculations to assert the reliability of these estimates.

## 3. Results

The current analysis comprised 5022 dentate adults aged 15+ years who were interviewed and orally examined. Of 39,651 persons eligible for interviews, 15,731 completed the interviews, resulting in an overall participation rate of 39.7% (15,731/39,651). Among 14,944 dentate persons who were interviewed, 5022 underwent oral examinations, yielding an overall examination participation rate of 33.6% (5022/14,944). Inter‐examiner reliability of examiners ranged from substantial to almost perfect agreement with intra‐class correlation coefficients for assessing periodontitis, DMFT, number of teeth missing due to pathology and number of teeth present, being 0.64, 0.96, 1.0 and 1.0, respectively. Self‐reported prevalence of chronic conditions in Australians aged 15+ years by their sociodemographic characteristics is shown in Table 1. Age was strongly associated with the prevalence of all chronic conditions except depression. Barring asthma, the prevalence of chronic conditions was significantly higher in Australians aged 45+ years than in their younger counterparts aged <45 years. While almost 7 in 10 younger adults, as opposed to nearly one third of older adults reported that they had no chronic conditions, the prevalence of multimorbidity was about five times higher in ≥45‐year‐olds (37.1%: 95% CI = 34.5, 39.7) compared with <45‐year‐olds (7.7%: 95% CI = 6.2, 9.5). Sex was associated with the prevalence of diabetes, asthma, arthritis/osteoarthritis and depression. Diabetes was more prevalent in males (9.3%: 95% CI = 7.7, 11.1) than females (5.8%: 95% CI = 4.6, 7.3), whilst females were more likely to have asthma, arthritis/osteoarthritis and depression than males. The proportion of males without any chronic condition (52.9%: 95% CI = 49.9, 55.9) was significantly higher than that of females (45.9%: 95% CI = 43.2, 48.6). The prevalence of asthma, depression and multimorbidity in Indigenous Australians was 30.2%, 26% and 41.1%, respectively, while the corresponding figures for their non‐indigenous counterparts were 13.1%, 11.8% and 23%, respectively. Australian adults living in rural/remote areas had significantly higher levels of heart diseases (10.7%: 95% CI = 8.8, 12.9 vs. 7%: 95% CI = 5.9, 8.4), high blood pressure (26.8%: 95% CI = 24.0, 29.8 vs. 21.6%: 95% CI = 19.6, 23.8), arthritis/osteoarthritis (25.0%: 95% CI = 22.2, 28.1 vs. 18.3%: 95% CI = 16.5, 20.2) and multimorbidity (28.2%: 95% CI = 25.5, 31.1 vs. 21.6%: 95% CI = 19.5, 23.8) than their counterparts dwelling in major cities. The prevalence of all chronic conditions except kidney disease and cancer was lower in individuals with a degree or higher qualification, while they were more likely to be without any chronic condition than their counterparts who did not have a degree or higher qualification (62.1%: 95% CI = 59.0, 65.1 vs. 44.3%: 95% CI = 41.8, 46.8). In contrast, multimorbidity was over two‐fold higher in adults who had a lower level of education (28.1%: 95% CI = 26.0, 30.3) than their counterparts with higher levels of education (12.0%: 95% CI = 10.3, 14.1). Income had an impact on the prevalence of all chronic conditions barring asthma, while people in the highest income category were 1.9 times more likely to be free of chronic conditions (62.4%: 95% CI = 58.9, 65.7 vs. 33.0%: 95% CI = 29.4, 36.8) and 3.7 times less likely to have multimorbidity (10.8%: 95% CI = 8.8, 13.2 vs. 40.0%: 95% CI = 36.7, 43.5) as opposed to their counterparts in the lowest income category.

**Table 1:** Self‐reported prevalence of chronic conditions in Australian adults aged 15 years and over by their sociodemographic characteristics.

Chronic conditions (CC)														
Sociodemographic characteristics	*n* (weighted%)	HD % (95% CI)	Diabetes % (95% CI)	KD % (95% CI)	Asthma % (95% CI)	HBP % (95% CI)	Stroke % (95% CI)	COPD %(95%CI)	A/O % (95% CI)	Cancer % (95% CI)	Depression % (95% CI)	No CC % (95% CI)	One CC % (95% CI)	≥2 CC % (95% CI)
Age (years)														
<45	2139 (46.4%)	**1.0 (0.6, 1.5)**	**2.4 (1.6, 3.8)**	**0.1 (0.0, 0.5)**	**16.1 (13.9, 18.5)**	**5.3 (3.9, 7.2)**	**0.2 (0.0, 0.7)**	**0.2 (0.1, 0.8)**	**3.1 (2.3, 4.2)**	**0.5 (0.2, 1.0)**	11.5 (9.6, 13.7)	**69.1 (65.9, 72.1)**	**23.2 (20.7, 26.1)**	**7.7 (6.2, 9.5)**
≥45	2882 (53.6%)	**14.5 (12.7, 16.6)**	**12.1 (10.4, 13.9)**	**1.4 (0.9, 2.2)**	**11.4 (9.9, 13.0)**	**38.5 (35.9, 41.1)**	**3.0 (2.3, 3.8)**	**2.1 (1.5, 2.9)**	**35.7 (33.1, 38.4)**	**4.5 (3.6, 5.7)**	12.7 (11.1, 14.5)	**32.6 (30.0, 35.2)**	**30.4 (28.1, 32.8)**	**37.1 (34.5, 39.7)**
Sex														
Male	2248 (51.3%)	9.4 (7.9, 11.2)	**9.3 (7.7, 11.1)**	1.1 (0.6, 1.9)	**10.4 (8.7, 12.4)**	25.1 (22.7, 27.7)	1.5 (1.1, 2.2)	1.1 (0.6, 1.8)	**14.1 (12.5, 16.0)**	2.9 (2.2, 3.8)	**9.1 (7.5, 10.9)**	**52.9 (49.9, 55.9)**	25.8 (23.3, 28.4)	21.3 (19.2, 23.6)
Female	2773 (48.7%)	6.6 (5.3, 8.2)	**5.8 (4.6, 7.3)**	0.5 (0.3, 1.0)	**16.9 (15.0, 19.0)**	20.9 (18.9, 23.0)	1.8 (1.3, 2.5)	1.3 (0.9, 2.0)	**26.5 (24.1, 29.0)**	2.4 (1.7, 3.2)	**15.5 (13.6, 17.5)**	**45.9 (43.2, 48.6)**	28.4 (26.0, 31.0)	25.7 (23.3, 28.3)
Indigenous identity														
Non‐indigenous	4936 (97.9%)	8.0 (7.0, 9.2)	7.4 (6.4, 8.6)	0.8 (0.5, 1.3)	**13.1 (11.8, 14.6)**	23.2 (21.5, 25.0)	1.6 (1.3, 2.1)	1.2 (0.8, 1.7)	20.1 (18.6, 21.7)	2.7 (2.2, 3.3)	**11.8 (10.6, 13.2)**	49.8 (47.7, 51.9)	27.2 (25.4, 29.0)	**23.0 (21.4, 24.7)**
Indigenous	84 (2.1%)	9.9 (4.2, 21.4)	16.0 (7.2, 31.6)	1.5 (0.2, 10.4)	**30.2 (17.2, 47.5)**	18.3 (10.1, 30.9)	2.9 (0.5, 14.9)	1.2 (0.2, 7.9)	22.2 (12.2, 36.9)	1.7 (0.3, 10.6)	**26.0 (13.3, 44.5)**	35.9 (22.5, 52.0)	23.0 (13.1, 37.1)	**41.1 (26.8, 57.1)**
Residential location														
Major cities	2969 (71.8%)	**7.0 (5.9, 8.4)**	7.3 (6.1, 8.8)	0.8 (0.5, 1.5)	13.2 (11.5, 15.1)	**21.6 (19.6, 23.8)**	1.5 (1.1, 2.1)	1.0 (0.7, 1.5)	**18.3 (16.5, 20.2)**	2.5 (1.9, 3.3)	11.5 (10.0, 13.2)	**51.4 (48.8, 54.1)**	27.0 (24.8, 29.3)	**21.6 (19.5, 23.8)**
Rural/remote	2052 (28.2%)	**10.7 (8.8, 12.9)**	8.3 (6.7, 10.1)	0.7 (0.4, 1.3)	14.5 (12.5, 16.8)	**26.8 (24.0, 29.8)**	2.0 (1.4, 2.9)	1.6 (0.9, 2.8)	**25.0 (22.2, 28.1)**	2.9 (2.1, 4.1)	14.0 (11.9, 16.5)	**44.5 (41.4, 47.7)**	27.3 (24.9, 29.8)	**28.2 (25.5, 31.1)**
Highest qualification														
Degree or higher	2026 (29.2%)	**4.3 (3.4, 5.5)**	**4.6 (3.4, 6.3)**	0.7 (0.2, 2.0)	**10.3 (8.7, 12.0)**	**15.8 (13.6, 18.3)**	**0.8 (0.5, 1.2)**	**0.3 (0.1, 0.7)**	**10.9 (9.3, 12.8)**	1.7 (1.1, 2.6)	**7.6 (6.2, 9.2)**	**62.1 (59.0, 65.1)**	25.8 (23.2, 28.6)	**12.0 (10.3, 14.1)**
Other/none	2930 (70.8%)	**9.7 (8.3, 11.2)**	**8.9 (7.6, 10.4)**	0.9 (0.6, 1.4)	**15.0 (13.3, 17.0)**	**25.8 (23.7, 28.0)**	**2.1 (1.6, 2.7)**	**1.5 (1.1, 2.1)**	**23.9 (21.9, 26.0)**	3.1 (2.4, 3.9)	**14.1 (12.4, 16.0)**	**44.3 (41.8, 46.8)**	27.6 (25.4, 29.9)	**28.1 (26.0, 30.3)**
Income tertile														
Lowest (<$40K)	1409 (33.6%)	**14.7 (12.2, 17.6)**	**13.4 (11.0, 16.1)**	**2.0 (1.1, 3.6)**	15.2 (12.7, 18.1)	**35.3 (32.0, 38.7)**	3.1 (2.2, 4.2)	**2.7 (1.9, 4.0)**	**36.3 (32.8, 40.0)**	**4.6 (3.5, 6.1)**	**18.3 (15.7, 21.1)**	**33.0 (29.4, 36.8)**	27.0 (23.9, 30.2)	**40.0 (36.7, 43.5)**
Middle ($40K –<$90K)	1289 (31.8%)	**8.1 (6.3, 10.3)**	**6.6 (4.9, 8.9)**	**0.4 (0.2, 1.0)**	11.1 (9.2, 13.4)	**21.2 (18.0, 24.9)**	1.8 (1.1, 3.0)	**0.6 (0.2, 1.6)**	**17.3 (14.9, 20.0)**	**2.2 (1.4, 3.3)**	**10.4 (8.3, 12.9)**	**52.6 (48.8, 56.3)**	26.7 (23.2, 30.5)	**20.7 (18.0, 23.8)**
Highest ($90K+)	1531 (34.6%)	**3.4 (2.4, 4.7)**	3.7 (2.5, 5.5)	**0.3 (0.1, 0.8)**	12.1 (10.1, 14.4)	15.5 (13.2, 18.2)	**0.2 (0.1, 0.5)**	0.5 (0.2, 1.2)	**9.0 (7.2, 11.2)**	1.3 (0.8, 2.2)	**6.5 (5.1, 8.3)**	**62.4 (58.9, 65.7)**	26.8 (23.8, 30.0)	**10.8 (8.8, 13.2)**

*Note*: 95% CI, 95% confidence intervals. Non‐overlapping 95% confidence intervals are highlighted in bold.

Abbreviations: A/O, arthritis/osteoporosis; COPD, chronic obstructive pulmonary disease; HBP, high blood pressure; HD, heart disease; KD, kidney disease.

### 3.1. Chronic Conditions and Tooth Loss

Table 2 shows that the mean number of missing teeth due to dental caries and periodontal disease was highest in Australian dentate adults with COPD (11.5), followed by those who had kidney disease (11.3), stroke (11.2) and heart disease (10.2). Individuals with one chronic condition (4.9: 95% CI = 4.4, 5.4) and multimorbidity (8.6: 95% CI = 7.9, 9.4) had lost more teeth by 1.9 and 3.3 times, respectively, compared with their counterparts who had no chronic conditions (2.6: 95% CI = 2.3, 2.9). Australian dentate adults with COPD had the highest prevalence of IND (45.1%) followed by people with kidney disease (44%), heart disease (33.6%), cancer (32.4%) and diabetes (31.3%). Compared with individuals who had no chronic conditions (4.1%: 95% CI = 3.2, 5.2), the prevalence of IND was almost 3.0 and 6.4 times higher in adults with one chronic condition (12.1%: 95% CI = 10.1, 14.5) and multimorbidity (26.3%: 95% CI = 23.1, 29.7), respectively.

**Table 2:** Mean number of missing teeth due to pathology^a^ per person and the prevalence of inadequate natural dentition^b^ (IND) in the Australian dentate adults aged 15 years and over by presence of chronic conditions.

Chronic condition	Number of missing teeth			Prevalence of IND		
	*n*	Mean	(95% CI)	*n*	Percentage	95% CI
Heart disease	401	10.2	(9.1, 11.3)	400	33.6	(27.6, 40.2)
Diabetes	393	9.1	(7.9, 10.3)	391	31.3	(25.1, 38.2)
Kidney disease	37	11.3	(8.5, 14.1)	37	44.0	(24.0, 66.1)
Asthma	665	4.0	(3.4, 4.7)	664	10.2	(7.7, 13.2)
High blood pressure	1256	8.7	(8.0, 9.4)	1252	25.0	(21.8, 28.4)
Stroke	104	11.2	(8.8, 13.6)	102	28.5	(19.2, 40.0)
Chronic obstructive pulmonary disease	63	11.5	(8.3, 14.7)	63	45.1	(30.7, 60.4)
Arthritis/osteoporosis	1092	9.4	(8.7, 10.2)	1088	27.2	(24.0, 30.7)
Cancer	157	9.8	(8.1, 11.5)	155	32.4	(23.4, 42.9)
Depression	590	5.2	(4.4, 6.0)	588	15.4	(11.9, 19.7)
No chronic condition	2345	**2.6**	**(2.3, 2.9)**	2340	**4.1**	**(3.2, 5.2)**
One chronic condition	1385	**4.9**	**(4.4, 5.4)**	1380	**12.1**	**(10.1, 14.5)**
≥2 chronic conditions	1292	**8.6**	**(7.9, 9.4)**	1287	**26.3**	**(23.1, 29.7)**

*Note*: 95% CI, 95% confidence intervals. Non‐overlapping 95% confidence intervals are highlighted in bold.

^a^Due to dental caries and periodontal disease.

^b^Inadequate natural dentition = presence of fewer than 21 natural teeth.

### 3.2. Chronic Conditions and Dental Caries

As per Table 3, Australian dentate adults with heart disease had the highest mean number of untreated DS per person (2.5), followed by individuals with depression (2.3), high blood pressure (1.7), stroke (1.6) and diabetes (1.5). Adults with multimorbidity displayed a significantly higher mean DS (2.0: 95% CI = 1.5, 2.6) than their counterparts with one chronic condition (1.1: 95% CI = 0.8, 1.3). Individuals with stroke had the highest mean number of DMFS: 60, followed by those with cancer (59.8), kidney disease (59.2), arthritis/osteoporosis (58.0) and heart disease (57.1). The increase in DMFS among adults with one chronic condition (33.4: 95% CI = 30.7, 36.1) and multimorbidity (51.3: 95% CI = 48.0, 54.5) was 1.7‐ and 2.6‐ fold, respectively, compared with their counterparts who had no chronic conditions (19.8: 95% CI = 18.3, 21.4).

**Table 3:** Mean number of decayed tooth surfaces per person (DS) and decayed, missing or filled tooth surfaces per person (DMFS) in the Australian dentate adults aged 15 years and over by presence of chronic conditions.

Chronic condition	DS			DMFS		
	*n*	Mean	(95% CI)	*n*	Mean	95% CI
Heart disease	401	2.5	(1.2, 3.7)	401	57.1	(52.1, 62.1)
Diabetes	393	1.5	(1.0, 2.1)	393	51.9	(46.7, 57.1)
Kidney disease	37	1.0	(0.0, 1.9)	37	59.2	(49.2, 69.3)
Asthma	665	1.3	(0.8, 1.9)	665	26.1	(22.8, 29.3)
High blood pressure	1256	1.7	(1.3, 2.1)	1256	51.9	(49.1, 54.6)
Stroke	104	1.6	(0.0, 3.2)	104	60.0	(53.0, 67.0)
Chronic obstructive pulmonary disease	63	0.6	(0.2, 1.0)	63	54.5	(41.0, 68.1)
Arthritis/osteoporosis	1092	1.3	(0.9, 1.7)	1092	58.0	(55.0, 61.0)
Cancer	157	1.4	(0.4, 2.5)	157	59.8	(53.3, 66.3)
Depression	590	2.3	(1.4, 3.3)	590	34.0	(29.9, 38.1)
No chronic condition	2345	1.4	(1.1, 1.7)	2345	**19.8**	**(18.3, 21.4)**
One chronic condition	1385	**1.1**	**(0.8, 1.3)**	1385	**33.4**	**(30.7, 36.1)**
≥2 chronic conditions	1292	**2.0**	**(1.5, 2.6)**	1292	**51.3**	**(48.0, 54.5)**

*Note*: 95% CI, 95% confidence intervals. Non‐overlapping 95% confidence intervals are highlighted in bold.

### 3.3. Chronic Conditions and Periodontal Disease

Australian dentate adults with diabetes had the highest prevalence of gingivitis (40.1%), followed by individuals with high blood pressure (33.9%), COPD (33.7%), stroke (33.6%) and depression (30.6%) (Table 4). The prevalence of gingivitis in adults who had no chronic conditions was 33.2% (95% CI = 30.0, 36.6); nevertheless, the prevalence of gingivitis among the three groups of adults who had no, one (30.2% 95% CI = 26.2, 34.5) or two or more (30.2%: 95% CI = 25.8, 35.1) chronic conditions was not significantly different. The prevalence of moderate or severe periodontitis (PI) was highest in people with heart disease (59.1%), followed by adults with cancer (56.7%), stroke (51.3%), diabetes (49.7%) and high blood pressure (48.5%). Individuals with one chronic condition (36.1%: 95% CI = 32.1, 40.2) and two or more chronic conditions (43.8%: 95% CI = 39.4, 48.3) showed significantly higher levels of PI than their counterparts with no chronic conditions (26.1%: 95% CI = 23.5, 28.9).

**Table 4:** Prevalence of gingival inflammation (GI) and moderate or severe periodontitis (PI) in the Australian dentate adults aged 15 years and over by presence of chronic conditions.

Chronic condition	Prevalence of GI			Prevalence of PI		
	*n* ^a^	Percentage	(95% CI)	*n* ^a^	Percentage	95% CI
Heart disease	69	25.3	(11.9, 45.9)	49	59.1	(39.3, 76.3)
Diabetes	286	40.1	(31.5, 49.4)	288	49.7	(41.9, 57.4)
Kidney disease	15	15.2	(3.3, 48.9)	14	35.3	(12.4, 67.6)
Asthma	584	30.0	(25.2, 35.3)	584	25.3	(20.7, 30.5)
High blood pressure	936	33.9	(29.3, 38.9)	932	48.5	(43.8, 53.2)
Stroke	59	33.6	(19.9, 50.8)	59	51.3	(36.8, 65.5)
Chronic obstructive pulmonary disease	41	33.7	(17.7, 54.5)	42	45.9	(28.1, 64.8)
Arthritis/osteoporosis	817	25.6	(21.3, 30.4)	815	47.7	(43.2, 52.4)
Cancer	105	24.7	(15.1, 37.7)	104	56.7	(43.2, 69.3)
Depression	501	30.6	(24.9, 37.1)	497	31.6	(26.4, 37.3)
No chronic condition	2284	33.2	(30.0, 36.6)	2290	**26.1**	**(23.5, 28.9)**
One chronic condition	1244	30.2	(26.2, 34.5)	1242	**36.1**	**(32.1, 40.2)**
≥2 chronic conditions	878	30.2	(25.8, 35.1)	870	**43.8**	**(39.4, 48.3)**

*Note*: 95% CI, 95% confidence intervals. Non‐overlapping 95% Confidence Intervals are highlighted in bold.

^a^ *n* was confined to participants without medical contraindications for periodontal probing.

## 4. Discussion

The present findings revealed that the prevalence of almost all selected chronic conditions, apart from asthma, was higher in adults aged 45+ years than in their younger counterparts. This was also reflected in the proportions of adults aged 45+ years with one chronic condition and multimorbidity, which were almost 1.3 and 5 times higher compared with the corresponding proportions of younger adults. These findings conform to those of previous studies in Australia [6, 16]. For instance, only 28% of people aged below 15 years were reported to have at least one chronic condition as opposed to 94% of Australian older adults aged 85+ years [23], while the mean age of people with asthma was reported to be less than 45 years [16]. The latter, in line with the current findings, indicates that asthma is likely to be more common among younger people than among their older counterparts. While the prevalence of diabetes was higher in males, females had higher levels of asthma, arthritis/osteoarthritis and depression than males—these findings corroborate with the previous reports [10]. Comparable with the ABS data [10], the present findings indicated that Australians residing in major cities were less likely to be affected by heart disease, high blood pressure, arthritis/osteoarthritis and multimorbidity than their equivalents in rural/remote Australia. Low education and income levels were associated with a high prevalence of almost all chronic conditions, except a few, as well as multimorbidity, which corroborated closely with the findings of the ABS [10]. Overall, our findings support the strong association of chronic conditions with age and a social gradient in the distribution of chronic conditions among Australian adults, as reported previously [6, 10, 11, 16].

The current findings with regard to chronic conditions and tooth loss indicated that Australian adults who had COPD and kidney disease were more likely to have lost their teeth, with both the mean number of missing teeth and the prevalence of IND being comparatively high among them. In contrast, a previous report based on NDTIS 2010 data showed that people with stroke had lost more teeth and had higher levels of IND compared with individuals who reported other chronic conditions [16]. However, in the NDTIS 2010 study, the adults with COPD were not included, and the participants were 18 years or older Australians, as opposed to the current study where the participants were Australians aged 15+ years. Importantly, our findings showed that tooth loss, denoted by the average number of missing teeth and the presence of IND, increased rapidly in Australian adults who had one chronic condition as well as multimorbidity compared with their counterparts with no chronic conditions. Previous studies indicated that people with chronic conditions were more likely to have unfavourable dental visiting behaviours such as not visiting a dental professional in the previous year and/or visiting due to a dental problem instead of a check‐up, which in turn could deteriorate their oral health and increase the risk for tooth loss [16, 17].

Whilst there is a lack of data at an Australian national level to compare with the findings from our study, Victorian adults who had attended a public dental service with chronic health problems such as diabetes, heart disease and arthritis were reported to have substantially higher levels of periodontal disease [24]. Similarly, our findings showed that people with diabetes, high blood pressure, heart disease and cancer had higher levels of gingivitis and periodontitis. Consistent with our findings, a nation‐wide study in the US reported that poor oral health was associated with diabetes, cardiovascular disease and mental health [25]. As mentioned hitherto, a recent umbrella review yielded associations between 28 chronic conditions and oral diseases that were supported by strong evidence [13]. For example, dental caries and tooth loss were associated with depression, dementia and cancer, while there were associations of periodontal disease with numerous chronic conditions, including diabetes, cardiovascular diseases, rheumatoid arthritis, chronic kidney disease, cancer and asthma. Likewise, we found that the dental caries experience was higher among people with heart disease, depression, stroke and cancer, whilst periodontal disease was associated with several chronic conditions, including diabetes, heart disease, high blood pressure and cancer. Evidence from the umbrella review showed that the individuals afflicted with chronic conditions were more likely to have oral diseases and/or being exposed to oral diseases, leading them to be at a higher risk for chronic conditions [13].

However, evidence derived from such studies should be interpreted with caution due to inherent limitations, including heterogeneity among the included systematic reviews, the predominance of observational data in meta‐analyses, and substantial variation in the diagnostic criteria used for oral diseases [8, 12, 13]. Accordingly, causality cannot be inferred from this body of evidence, although the reported associations may operate bidirectionally. For instance, diabetes and periodontitis show bidirectional relationships—whilst people with periodontitis are more likely to have poorly controlled diabetes and a higher risk of developing diabetes, the risk of periodontitis is greater in people with diabetes [8]. The current classification of periodontal disease considered diabetes‐associated periodontitis as a modifying factor for periodontitis rather than a distinct disease [26]. Associations between periodontitis and rheumatic arthritis have been reported, whereas the treatment of periodontitis has lowered the different parameters that assess rheumatic arthritis, such as rheumatoid factor (RF) seropositivity and anti‐citrullinated proteins autoantibodies (ACPAs) [27–29]. Additionally, people with rheumatic arthritis are more likely to be edentulous and affected by periodontitis than people without rheumatic arthritis [30] as it may limit a person’s physical ability to maintain oral health [8]. Likewise, our finding that individuals with stroke had the highest mean DMFS may also support this fact—stroke patients are more likely to present with impaired manual dexterity, which in turn can compromise their oral health.

Apart from being strongly associated with major chronic conditions and causing disability, poor oral health shares common risk factors with chronic conditions [8, 12, 24]. For example, risk factors such as smoking, alcohol use, diet, being overweight, poor hygiene, stress and trauma are in general common to oral diseases and numerous chronic conditions, including diabetes, heart diseases, cancer and stroke. Shared pathways mediated through these common risk factors may underlie the observed associations between oral diseases and chronic conditions, and therefore, mitigating the effects of such upstream factors is pivotal for the prevention and control of both oral diseases and chronic conditions [8, 12]. From a broad public health perspective, this reinforces the importance of common risk factor approaches within the well‐established shared‐risk framework. Moreover, the shared risk factor profile between oral diseases and other chronic conditions increases the likelihood of their co‐occurrence [8]. This may contribute to the clustering of disease burdens, where oral diseases and chronic conditions co‐exist, particularly among older adults, as indicated by the current findings. Such clustering has important implications for public health, highlighting the need for integrated preventive approaches that address common risk factors rather than isolated chronic conditions [8, 12].

On the other hand, there may be a potential for chronic conditions as well as medications used to treat them to initiate or aggravate oral health conditions [24]. For instance, diabetic patients are more susceptible to developing oral candidiasis, whereas individuals on high blood pressure/anti‐epileptic medications may have an increased risk of developing dry mouth. The oral microbiome, its by‐products and their interplay with the host immune system are regarded to be the key biological pathway that explains oral–chronic disease relationships [13]. An array of evidence suggests that associations with oral diseases, particularly periodontal disease, and a myriad of chronic conditions can be attributed to bacteraemia and systemic inflammation, which is mediated through the pathogenic microbes and their by‐products accessing the blood stream. Concomitant low‐grade inflammation has been responsible for the development of a wide range of chronic conditions discussed hitherto [13]. Moreover, such a status of inflammation may not only cause changes to lipid metabolism but also increase glucose intolerance as well as insulin resistance, progressing to heart diseases, diabetes and other metabolic disorders [31, 32]. In a recent joint workshop organised by the European Federation of Periodontology (EFP) and the World Heart Federation (WHF), the evidence base for associations between periodontitis and cardiovascular diseases and the mechanisms underpinning such associations were updated [33]. According to the resulting consensus report, several biological mechanisms may explain these epidemiological associations. These include a higher incidence of bacteraemia in patients with periodontitis following routine oral functions (e.g., tooth brushing/flossing) or dental procedures (e.g., tooth extraction/scaling), the presence of oral bacteria within atheroma lesions, and the promotion of atheroma formation by periodontal pathogens and bacterial products. These mechanisms may be further compounded by the increased production of systemic inflammatory mediators such as C‐reactive protein (CRP), as well as pro‐inflammatory cytokines including serum interleukin (IL)‐6 and tumour necrosis factor (TNF)‐α.

On the other hand, nutrition and social determinants, also regarded as modifiable factors in oral–chronic disease relationships, can mediate these associations through a non‐biological shared pathway [34, 35]. While nutrition plays a major role in metabolic disorders such as diabetes mellitus and obesity, dietary patterns are gravely affected by poor oral health, which, in turn, has a significant impact on such metabolic disorders [13, 34]. Furthermore, social determinants, including low levels of education as well as income and disadvantaged socioeconomic status, may act as potential confounders and/or mediators of the oral–chronic disease associations [25], as reflected by the current findings. Additionally, regarding the role of prevention, other factors, such as the use of remineralising agents like casein [36] and zinc hydroxyapatite [37], should be considered in future studies that explore oral–chronic disease relationships.

Whilst supporting the associations between oral and chronic conditions, our findings point to the importance of promoting oral care and integrating it into the overall health care of people with chronic conditions. Such a holistic approach is particularly relevant given that people who have chronic conditions are inclined to have undesirable dental visiting patterns and be living with a double detriment of compromised general health as well as oral health, eventually leading to poor levels of general health‐ and oral health‐related quality of life [16, 17]. Moreover, physical health impacts that can arise from chronic conditions such as stroke and rheumatoid arthritis may act as a barrier for them to access oral health care. As the current findings disclosed, there was a multifold increase in the risk of poor oral health across the groups, from people with one chronic condition to those individuals with multimorbidity in comparison to people with no chronic conditions. More importantly, social inequality in the distribution of both chronic diseases and oral diseases has been on the incline in Australia, with socioeconomically disadvantaged people increasingly affected by poor general health and oral health conditions [11, 38, 39]. This situation has been further aggravated with Medicare, the national scheme in Australia providing free or subsidised health services, not covering the cost of most dental services, while nearly two‐thirds of Australians being ineligible to receive publicly funded dental care [39]. In this context, it was noteworthy that Australia introduced an Enhanced Primary Care Dental Scheme (EPCDS) in 2004, providing $220/year for patients with chronic diseases to seek oral care, which was subsequently increased to $4250 over 2 years with the introduction of the Chronic Disease Dental Scheme (CDDS) in 2007 [37, 38]. However, CDDS was abolished in 2012, and no alternative scheme to provide oral care for people with chronic conditions has been established since [38, 39]. Abolition of CDDS might have potentially contributed to increasing the burden of oral disease among patients with chronic systemic conditions. Although ascertaining such contributions was beyond the scope of our study, it would be worthwhile to explore this in future work.

This study has some major strengths, including using rigorous epidemiological survey methods to recruit a sample of adults who were representative of Australians and employing a uniform examination scheme. In addition, the quality control of the study, as indicated by a high level of inter‐examiner reliability and agreement, was ensured by providing adequate training to interviewers and oral examiners. This study has several limitations. First, its cross‐sectional and descriptive design precludes any inference of causal relationships. Second, the absence of multivariable analysis was one of the most important limitations of the study. The analysis was based solely on descriptive statistics; therefore, important confounding factors, such as age, smoking habits, socioeconomic status and frequency of dental visits, were not simultaneously controlled for using multiple regression models. As age is strongly associated with both systemic chronic conditions and oral conditions such as edentulism and periodontitis, the reported associations may have been substantially confounded by age. By including only dentate adults, the study may have introduced selection bias and underestimated the strength of the association between systemic chronic conditions and tooth loss, as edentulous individuals who were excluded from the analysis might have poorer oral and general health than dentate participants. Furthermore, the diagnosis of chronic conditions was based on self‐report questionnaire data rather than medical records, which may have introduced a recall bias or misclassification due to participants being unaware of their condition. Additional limitations include relatively low participation rates and the restricted availability of information on chronic conditions for orally examined dentate participants, both of which may have introduced a selection bias. Furthermore, the partial recording protocols used in the present study (where gingival inflammation was assessed on only six index teeth) may have underestimated the prevalence of gingivitis. Future studies can address this limitation by replacing the partial recording system of gingivitis with a full assessment of gingivitis in all teeth present, except third molars.

## 5. Conclusions

The findings suggested that age was strongly associated with chronic conditions in Australian dentate adults, and the distribution of chronic conditions among them was substantially impacted by social inequality. Moreover, our findings indicated that the overall oral health status, as indicated by tooth loss as well as dental caries and periodontal disease levels, was worse in Australian dentate adults with chronic conditions than that in those without. Among groups defined by the number of chronic conditions (none, one, and ≥2), oral health status was worst in individuals with multimorbidity. In the absence of a specific scheme targeting to cater the oral health needs of Australians with chronic conditions, our findings emphasise the importance of planning and implementing sustainable oral health programmes in promoting oral care and integrating them into the overall health care of Australians with chronic conditions to improve their quality of life.

**Nomenclature**
AAP: American Academy of Periodontology
ABS: Australian Bureau of Statistics
AIHW: Australian Institute of Health and Welfare
ASIR: Age‐standardised incidence rate
CATI: Computer‐assisted telephone interview
CDC: Centers for Disease Control and Prevention
COPD: Chronic obstructive pulmonary disease
DHS: Australian Government Department of Human Services
DMFS: Decayed, missing or filled tooth surfaces
DMFT: Decayed, missing or filled teeth
DS: Decayed tooth surfaces
EFP: European Federation of Periodontology
GI: Gingival inflammation
IND: Inadequate natural dentition
NHANES: National Health and Nutrition Examination Survey
NSAOH: National Study of Adult Oral Health
PI: Moderate or severe periodontitis
WHF: World Heart Federation
WHO: World Health Organization.

## Author Contributions

**Najith Amarasena**: conceptualisation (lead), formal analysis (supporting), writing – original draft (lead), writing – review and editing (equal). **Manisha Tamrakar, Gloria Mejia and Liana Luzzi**: conceptualisation (supporting), writing – original draft (supporting), writing – review and editing (equal). **Sergio Chrisopoulos**: conceptualisation (supporting), formal analysis (lead), writing – original draft (supporting), writing – review and editing (equal).

## Funding

The original study (NSAOH 2017−18), on which the present article was based, was funded by the National Health and Medical Research Council (Grant 1115649). Open access publishing facilitated by Adelaide University, as part of the Wiley–Adelaide University agreement via the Council of Australasian University Librarians.

## Disclosure

All authors have read and approved the final version of the manuscript. Najith Amarasena and Sergio Chrisopoulos had full access to all of the data in this study and take complete responsibility for the integrity of the data and the accuracy of the data analysis.

## Ethics Statement

NSAOH was reviewed and approved by The University of Adelaide’s Human Research Ethics Committee (HREC; H‐2016‐046).

## Consent

Interviewed subjects provided verbal consent prior to answering questions. Parental/guardian consent was obtained for participants aged 15–17 years. All examined subjects provided signed informed consent prior to the examination (parents/guardians of participants aged 15–17 years provided signed informed consent prior to the examination).

## Conflicts of Interest

The authors declare no conflicts of interest.

## Practical Application

The present findings, based on the latest national oral health data, may assist clinicians to recognise patients with chronic conditions who are at an increased risk for oral conditions and manage them accordingly.

## Data Availability

The datasets used during the current study are available from the corresponding author via the completion of a data request.
